# Clinical features and long-term prognosis of acute fibrinous and organizing pneumonia histologically confirmed by surgical lung biopsy

**DOI:** 10.1186/s12890-022-01852-z

**Published:** 2022-02-08

**Authors:** Min Chul Kim, Yeon Wook Kim, Byoung-Soo Kwon, Junghoon Kim, Yeon Joo Lee, Young-Jae Cho, Ho Il Yoon, Jin-Haeng Chung, Jae Ho Lee, Choon-Taek Lee, Jong Sun Park

**Affiliations:** 1 Division of Pulmonary and Critical Care Medicine, Department of Internal Medicine, Seoul National University Bundang Hospital, 82, Gumi-ro 173 Beon-gil, Bundang-gu, Seongnam-si, Gyeonggi-do 13620 Republic of Korea; 2 Department of Internal Medicine, Seoul National University College of Medicine, Seoul, Republic of Korea; 3 Division of Pulmonology, Allergy and Critical Care Medicine, Department of Internal Medicine, Yongin Severance Hospital, Yonsei University College of Medicine, Yongin, Republic of Korea; 4 Department of Radiology, Seoul National University Bundang Hospital, Seongnam, Republic of Korea; 5 Department of Pathology and Translational Medicine, Seoul National University College of Medicine, Seongnam, Korea

**Keywords:** Acute fibrinous and organizing pneumonia, Interstitial lung disease, Mortality, Prognosis, Survival

## Abstract

**Background:**

Acute fibrinous and organizing pneumonia (AFOP) is a rare interstitial pneumonia characterized by intra-alveolar fibrin deposition and organizing pneumonia. The clinical manifestations and long-term prognosis of AFOP are unclear. Our objective was to investigate the clinical features and prognosis of AFOP.

**Methods:**

We identified patients diagnosed with AFOP by surgical lung biopsy between January 2011 and May 2018 at Seoul National University Bundang Hospital. We retrospectively reviewed clinical and radiologic findings, treatment, and outcomes of AFOP.

**Results:**

Fifteen patients with histologically confirmed lung biopsies were included. The median follow-up duration was 2.4 (range, 0.1–82) months. The median age was 55 (range, 33–75) years, and four patients were immunocompromised. Fever was the most common clinical presentation (86.7%). Patchy ground-glass opacities and/or consolidations were the most predominant findings on chest computed tomography images. Nine patients (60%) received mechanical ventilator care, and eight patients (53.3%) died. The non-survivors tended to have slightly higher body mass index (BMI) and a long interval between symptom onset and diagnosis than the survivors, but these findings were not statistically significant. Among seven survivors, five patients were discharged without dyspnea and oxygen supplement.

**Conclusions:**

The clinical course of AFOP was variable. Although AFOP was fatal, most of the patients who recovered from AFOP maintained normal life without supplemental oxygen therapy and respiratory symptoms.

## Introduction

Acute fibrinous and organizing pneumonia (AFOP) is a rare interstitial pneumonia that is histologically characterized by intra-alveolar fibrin deposition and organizing pneumonia. It was first described in 2002 by Beasley et al. as an unusual type of acute lung injury [1], and it was recognized as a new rare idiopathic interstitial pneumonia in the 2013 American Thoracic Society/European Respiratory Society statement [2]. Although AFOP is categorized as idiopathic interstitial pneumonia [3–5], variable conditions including autoimmune diseases, infections, exposure to some drugs, hematologic disorders, and occupational exposures have been associated with AFOP according to several case reports or case series [6–10]. Furthermore, there are few studies on the clinical characteristics and outcomes of AFOP. Therefore, we investigated the clinical characteristics, radiologic findings, and prognosis of AFOP, and investigated the prognostic factors of AFOP.

## Material and methods

### Study design and patient characteristics

We identified patients diagnosed with AFOP by surgical lung biopsy between January 2011 and May 2018 at Seoul National University Bundang Hospital, a tertiary teaching hospital. We retrospectively reviewed their demographics, clinical features, radiologic findings, treatments, and outcomes based on electronic medical records. We collected data based on the electrical medical record of patient’s symptom and physician’s examination. Laboratory findings were collected from the results at the presentation of symptoms. Results of chest computed tomography (CT) images, bronchoalveolar larvage (BAL) fluid analysis, pulmonary function test (PFT) were collected from the first data performed after the onset of symptoms. Chest CT findings were analyzed by an experienced pulmonary radiologist. The CT findings were classified into three categories. If the CT image showed multifocal patchy or diffuse ground-glass opacity (GGO) or consolidations, they classified into the GGO/Consolidation dominant type. CT findings with multiple ill-defined solid nodules or centrilobular tiny nodules with less GGO/consolidations were classified into nodular dominant type. And CT findings with band-like fibrosis, architectural distortion and volume loss were classified into fibrosis dominant type.

All cases were histologically confirmed by surgical lung biopsy. The tissue samples showed intra-alveolar fibrinous aggregation and organizing pneumonia pattern with no evidence of hyaline membrane, granulomatous inflammation, conspicuous eosinophils, extensive bronchopneumonia or abscess formation were reported to consistent with AFOP [1].

To exclude an infectious cause, we examined quantitative microbiological cultures, mycobacterial cultures, respiratory virus polymerase chain reaction (PCR), and pneumocystis jirovecii PCR in sputum or BAL fluid samples. Echocardiography was performed to exclude heart failure and pulmonary edema. All cases were discussed at our multidisciplinary team discussion which included pulmonologists, thoracic surgeon, pulmonary radiologist, and pulmonary pathologist.

### Statistical analysis

Data are presented as the median and range. Continuous variables were compared using the Mann–Whitney U‑test. Categorical variables were compared using a Fisher's exact test. A P-value < 0.05 was considered statistically significant. The data about patient’s survival time were presented by Kaplan–Meier survival curve. SPSS statistics version 21 software was used for statistical analysis.

## Results

### Baseline characteristics

**Fig. 1:**
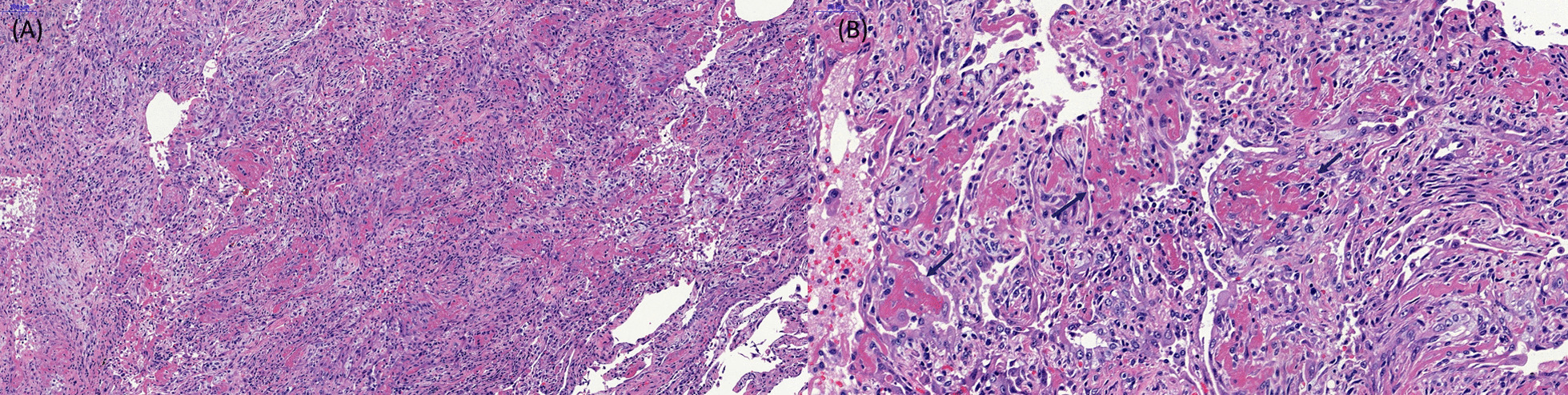
Representative histological features of acute fibrinous organizing pneumonia (AFOP). **a** The surgical lung biopsy specimen showed diffuse filling of alveolar spaces by fibrin balls associated with organizing pneumonia, interstitial lymphocytes and myxoid interstitial expansion (hematoxylin and eosin [H&E] stain, × 100). **b** The high power-view of the specimen showed intra-alveolar fibrin aggregates (arrow) which are characteristic findings of AFOP. Interstitial lymphoplasma cell infiltration, fibroblast proliferation with organization, and enlarged type II pneumocytes are present. Hyaline membranes, significant neutrophils or eosinophils are absent. (hematoxylin and eosin [H&E] stain, × 250)

A total of 15 patients were histologically diagnosed with AFOP by surgical lung biopsy. Representative microscopic images are shown in Fig. 1. Ten patients were men, and five were women, with a median age of 55 (range, 33–75) years. Seven patients were never smokers, and none had a previous history of lung disease. However, four patients showed underlying emphysema on chest CT image. One patient showed underlying chronic inflammatory sequelae, presumed to be pulmonary tuberculosis, and four patients were immunocompromised.

Three patients underwent allogeneic peripheral blood stem cell transplants for acute myeloid leukemia before the diagnosis of AFOP. One patient had a kidney transplant for immunoglobulin A nephropathy and was taking cyclosporin, mycophenolate, and deflazacort. Five out of fifteen patients were immunocompetent and did not have underlying disease such as diabetes, hypertension, heart disease, and lung disease. And their CT images showed no signs of underlying lung disease, such as emphysema or old inflammatory sequelae.

The median time from onset of symptoms to diagnosis was 17 (range 6–62) days. The most common clinical presentation was fever (86.7%), followed by cough (80%) and dyspnea (73.3%). Sputum production was relatively uncommon (40.0%). C-reactive protein (CRP) level was elevated above 0.5 mg/dL in 14 patients. In contrast, procalcitonin level was normal in seven patients, and none had a procalcitonin level > 2.0 ng/dL. Lactate dehydrogenase (LDH) was measured in eight patients, of which two were in the normal range and six were elevated. Among 13 patients who underwent bronchoalveolar lavage, seven patients showed a neutrophil-dominant cellular pattern. One patient showed a lymphocyte-dominant cellular pattern, and two patients showed mixed cellularity of both elevated lymphocytes and neutrophils (Table 1).

**Table 1:** Baseline characteristics of the study patients

Variables	N = 15
**Patients demographics**	
Age, years	55 (33–75)
Sex, male	10 (66)
BMI, kg/m^2^	22.3 (18.3–27.7)
Smoking (N = 13)	
Non-smoker	7/13 (53.8)
Ever-smoker	6/13 (46.2)
Pack year, years	13.8 (5.0–35.0)
**Underlying diseases**	
DM	2 (13.3)
HTN	7 (46.7)
Heart disease	3 (20.0)
Kidney transplantation	1 (6.7)
Bone marrow transplantation	3 (20.0)
**Clinical presentations and vital signs**	
Fever	13 (86.7)
Dyspnea	11 (73.3)
Cough	12 (80.0)
Sputum	6 (40.0)
SpO_2_, % (N = 14)	90.5 (68–98)
PaO_2_, mmHg (N = 8)	54.6 (40.6–72.2)
**Laboratory findings**	
WBC, × 10^3^/μl	8.21 (0.96–16.40)
CRP, mg/dL	9.74 (0.30–28.33)
Procalcitonin, ng/mL (N = 13)	0.26 (0.05–1.69)
LDH, IU/L (N = 8)	484.5 (167–845)
**Bronchoalveolar lavage fluid cell analysis (N = 13)**	
WBC, /μl	200 (4–900)
Neutrophil, %	30.0 (3–93)
Lymphocyte, %	12.5 (3–55)

Data are presented as n (%) or median (range)

**Fig. 2:**
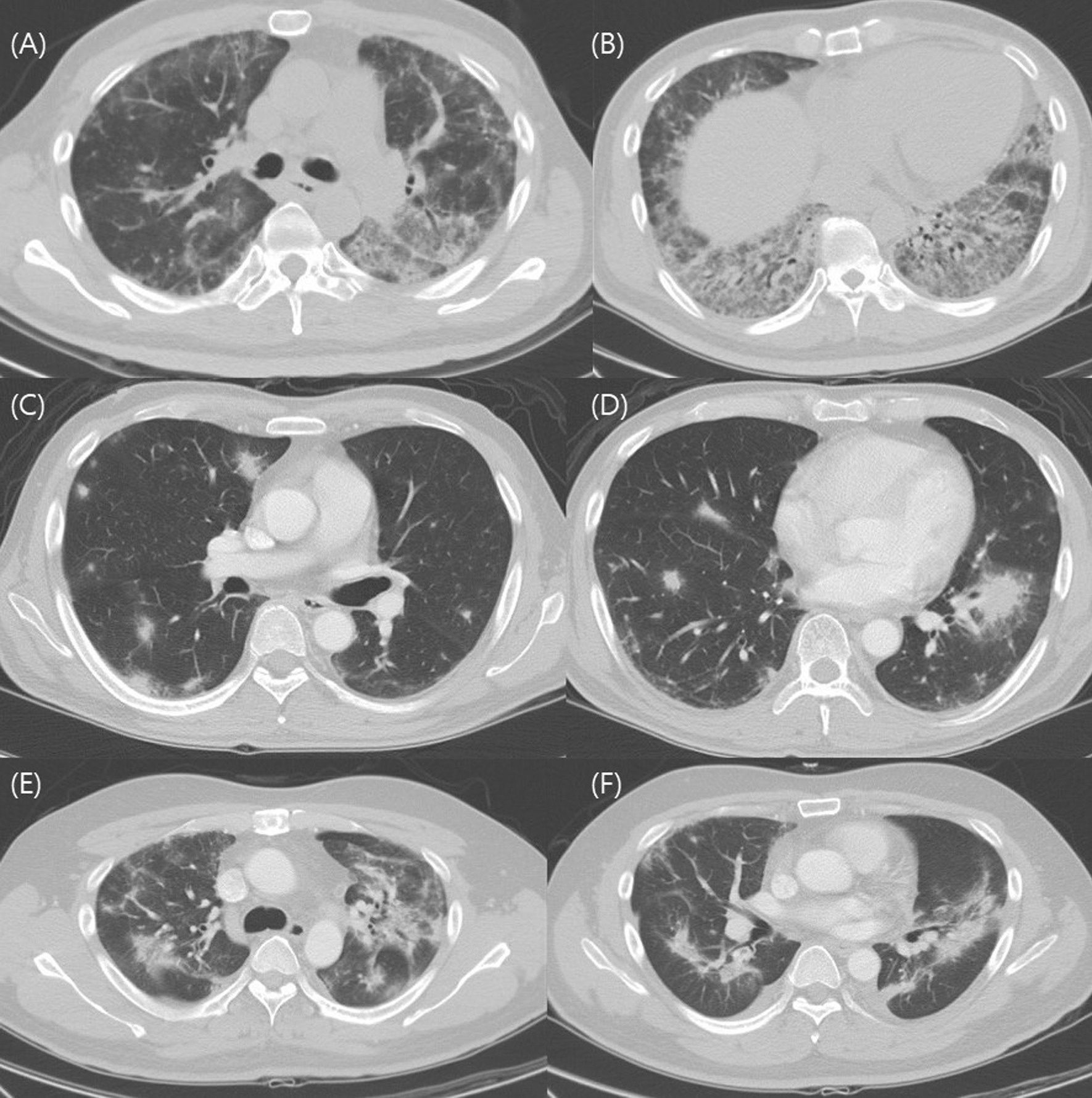
Representative CT images of acute fibrinous organizing pneumonia (AFOP). CT images A and B are from a 45-year-old man which show patchy and diffuse ground-glass opacity(GGO), peribronchovascular consolidation (GGO/consolidation dominant type). CT image C and D are from a 52-year-old man and show ill-defined nodular opacities in both lungs (nodule dominant type). CT image E and F are from a 40-year-old man and show fibrotic band at both lungs with diffuse GGO/consolidation (fibrosis dominant type). In figure F, small amount of bilateral effusion is seen

We analyzed chest CT findings of the study patients. Of nine patients who showed GGO/consolidation, five showed patchy or diffuse distribution, and four showed subpleural and peribronchial distribution. Two patients showed a fibrotic pattern. Five patients showed upper lobe predominancy, and six patients showed lower lobe predominancy. Most of the patients (86.7%) showed bilateral lung involvement. Eleven patients showed pleural effusions without significant hypoalbuminemia or evidence of heart failure. Thoracentesis and pleural fluid analysis were performed in five patients. Among them, two patients showed polymorphonuclear cell-dominant exudate, and other two patients showed mononuclear cell-dominant exudate. One patient also showed exudate, but the differential counting could not be evaluated because of the severe cell degeneration of the sample. In the rest of the patients, the amount of pleural effusion was too small to consider thoracentesis. (Table 2, Fig. 2).

**Table 2:** CT findings of the study patients

Variables	N = 15
**Patterns**	
GGO/consolidation dominant type	9 (60.0)
Nodule dominant type	4 (26.7)
Fibrosis dominant type	2 (13.3)
**Right and left predominancy**	
Right lung predominant	1 (6.7)
Left lung predominant	1 (6.7)
Symmetric	13 (86.7)
**Upper and lower predominancy**	
Upper lobe predominant	5 (33.3)
Lower lobe predominant	6 (40)
No definite predominance	4 (26.7)
**Pleural effusion**	11 (73.3)
Unilateral	0 (0)
Bilateral	11 (73.3)

Data are presented as n (%)

### Treatment and outcomes

All patients were treated with intravenous corticosteroids. The initial steroid dose was median 1.2 mg/kg (range, 0.7–1.4), and seven patients underwent steroid pulse therapy; three patients received additional immunosuppressants such as azathioprine, infliximab, rituximab, and cyclophosphamide because lung lesion did not improve after steroid pulse therapy (Table 3).

**Table 3:** Treatment of the study patients

Variables	N = 15
**Steroids**	
Initial dose*, mg/kg	1.2 (0.7–1.4)
Duration of ≥ 0.5 mg/kg of steroids, day	22 (11–49)
Pulse therapy	7 (46.7)
**Immunosuppressants**	
Azathioprine	1 (6.7)
Infliximab	1 (6.7)
Rituximab plus cyclophosphamide	1 (6.7)
**Lung transplantation**	1 (6.7)

Data are presented as n (%) or median (range)

*Prednisolone equivalent dose

Eleven patients (73.3%) were admitted to the intensive care unit for a median of 12 days due to respiratory failure. Among them, nine patients (81.8%) received mechanical ventilator care. Of the nine patients who received mechanical ventilator care, one patient was applied pressure support mode ventilation, and the remaining eight patients all received pressure-controlled ventilation. These eight patients showed decreased dynamic compliance of median 20.1 ml/cmH_2_O (range 13.6–29.2), and the PaO_2_/FiO_2_ ratio (PFR) after intubation was less than 100 in two patients, 100 to 200 in three patients, and 200 to 300 in two patients. The other one patient was not able to measure PFR due to immediate ECMO apply. Three patients (27.3%) received venovenous extracorporeal membrane oxygenation (VV-ECMO), and one patient received bilateral lung transplantation.

**Fig. 3:**
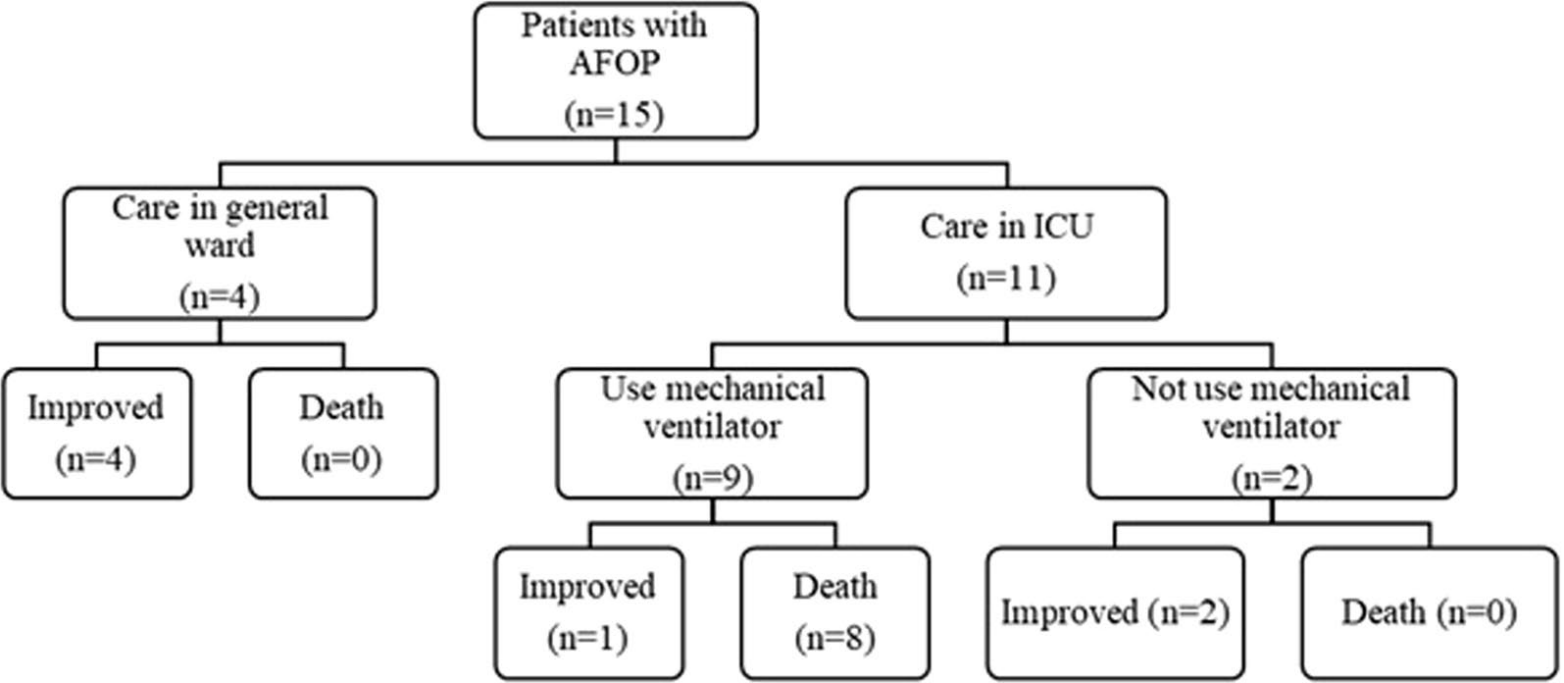
Clinical course of 15 patients with acute fibrinous organizing pneumonia (AFOP)

Eight patients died, including the three patients who received VV-ECMO and the one who received lung transplantation. Although in-hospital mortality was 53.3%, the five patients who survived were discharged without dyspnea and oxygen supplement. Two patients were transferred or discharged with long-term oxygen therapy (Table 4, Fig. 3). To find predictors for mortality, we compared clinical characteristics between survivors and non-survivors.

**Table 4:** Outcomes of the study patients

Variables	N = 15
ICU admission	11 (73.3)
Mechanical ventilator care	9 (60)
VV-ECMO apply	3 (20)
Length of ICU stay, days	12 (5–23)
Length of hospital stay, days	23 (12–75)
In-hospital mortality	8 (53.3)

Data are presented as n (%) or median (range)

*ICU* intensive care unit, *VV-ECMO* veno-venous extracorporeal membrane oxygenation

**Fig. 4:**
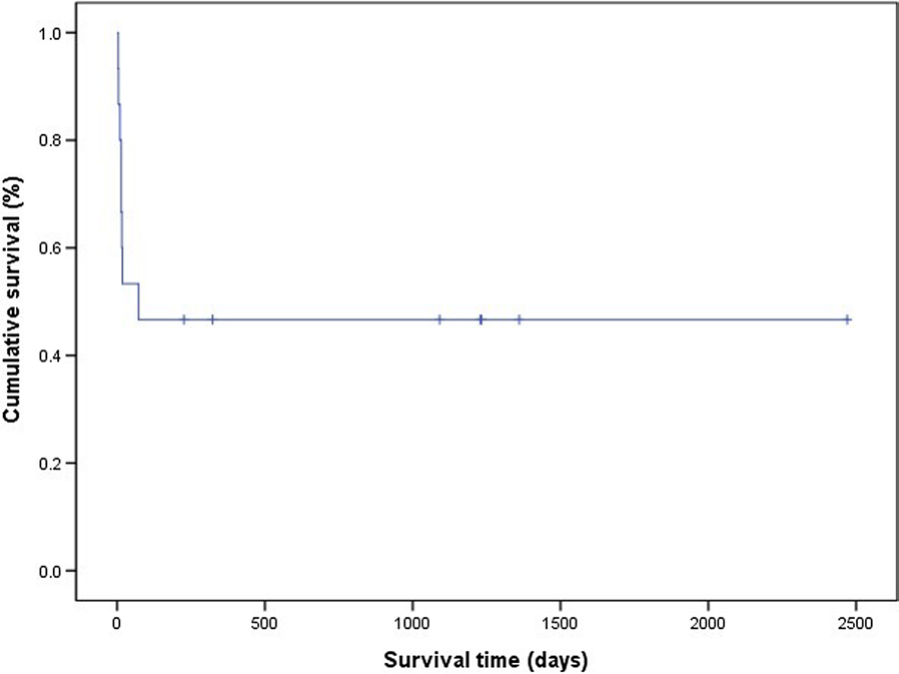
Kaplan–Meier survival plot of the study patients

The non-survivors tended to have slightly higher body mass index (BMI) and longer interval between symptom onset and diagnosis than the survivors, but these findings were not statistically significant. The underlying immunocompromised state was not associated with mortality. Analysis of the outcomes according to the main radiologic type revealed that patients with GGO/consolidation dominant radiologic findings tended to show the highest mortality, but it was not statistically significant. BAL fluid cellular pattern was not different between survivors and non-survivors (Table 5). We followed seven survivors for a median of 40 months (range, 7–81 months), and most survived for a long period without recurrence (Fig. 4). Only one patient had a recurrence of AFOP after reducing corticosteroid dose during the 3-year follow-up period after discharge.

**Table 5:** Comparisons between the survivor and non-survivor groups

	Survivor group (N = 7)	Non-survivor group (N = 8)	*p*-value
**Demographics**			
Age, years	52.0 (40–78)	56.0 (33–77)	0.867
Sex, male	5 (71.4)	5 (62.5)	1.000
Never smoker	3 (42.9)	4/6 (57.1)	0.592
BMI, kg/m^2^	21.6 (18.3–27.7)	22.9 (20.9–24.8)	0.189
Immunocompromised state	3 (42.9)	1 (12.5)	0.282
**Presentations and vital signs**			
Time from symptom onset to diagnosis	16 (6–26)	23 (7–62)	0.189
Presence of fever	5 (71.4)	8 (100)	0.200
SpO_2_, %	91 (68–98)	90 (77–95)	0.535
PaO_2_, mmHg	59.8 (47.8–72.2)	46.5 (40.6–65.7)	0.343
**Laboratory findings**			
WBC, × 10^3^/μL	8.40 (4.30–12.30)	7.31 (0.96–16.40)	1.000
CRP, mg/dL	10.50 (0.30–24.60)	9.34 (1.10–28.30)	0.779
Procalcitonin, ng/mL	0.19 (0.07–0.90)	0.52 (0.05–1.69)	0.445
BAL fluid cell analysis*			
Neutrophil dominant pattern	4/6 (66.7%)	3/6 (50.0%)	1.000
**Radiologic findings**			
GGO/consolidation dominant type	3 (42.9%)	6 (75.0%)	0.386
Nodule dominant type	2 (28.6%)	2 (25.0%)	
Fibrosis dominant type	2 (28.6%)	0 (0.0%)	

Data are presented as n (%) or median(range)

*p*-value: Statistical significance test was done by Mann–Whitney U-test or Fisher’s exact test

^*^Bronchoalveolar lavage (BAL) was done in 13 patients. One sample had few nucleated cells, so BAL fluid cell analysis were available in six out of seven survivors and six out of eight non-survivors

## Discussion

We analyzed the clinical characteristics and long-term prognoses of 15 patients with AFOP. The mortality of AFOP was 53.3%, but most of the patients who survived had normal life without respiratory sequelae that require long-term oxygen supplement over a median of 40 months of follow-up.

Several studies reported characteristics of AFOP [1, 9, 10]. However, those studies included AFOP diagnosed by percutaneous needle biopsy (PCNB) or endobronchial ultrasound-guided transbronchial lung biopsy (TBLB). However, all our AFOP patients were diagnosed with surgical lung biopsy, so the diagnostic confidence was high. Moreover, lung biopsies are carefully reviewed by a specialized lung pathologist, and secondary causes were thoroughly evaluated.

The most common clinical presentation of AFOP was fever, and the dominant radiologic pattern was a diffuse or patchy distribution of GGO and consolidation. Therefore, it is difficult to differentiate AFOP from pneumonia at the initial presentation. In laboratory findings, CRP was elevated in most patients with AFOP, but the elevation of procalcitonin level was relatively rare compared to elevation of CRP.

The time from initial symptom to diagnosis was variable, ranging from 6 to 26 days. Most patients started antibiotics when pneumonia was suspected for the first time. After confirmation of no response to antibiotics, a surgical lung biopsy was performed. Thus, there was a delay in the diagnosis of AFOP. Time from symptom onset to diagnosis tended to be longer in the non-survivor group than in the survivor group. However, it was not statistically significant.

Similar to other studies, some patients were associated with immunologic problem or environmental toxic chemicals [6, 9, 10]. Three patients had a history of bone marrow transplantation and one patient had a history of kidney transplantation in our study. Two patients finally proved to have an association with a specific humidifier disinfectant, which was known to have caused toxic lung injury in South Korea between 1995 and 2011 [11, 12]. However, survival was not different according to the presence of associated causes or immunocompromised status.

In our study, the overall mortality rate was 53.3%, which was similar to those of previous observational studies (52.9% [1] and 53.8% [10]). One study [9] showed a much lower mortality rate than these studies. Two out of 20 patients died due to a cause other than AFOP, and all other patients survived. Recently Onishi et al., reported the mortality rate of AFOP as 6% [13]. AFOP could have heterogenous phenotype and further large-scaled study would be needed to better understand the clinical course of AFOP.

Non-survivors tended to have high body mass index, long interval between symptom onset and diagnosis compared to survivors. However, those factors were not statistically significant due to the small number of patients. Further studies, including more patients, would be needed to find the prognostic factors.

Our study has several strengths. First, the patients were all diagnosed by surgical lung biopsy, which increases the diagnostic confidence. Second, the CT images and lung histological specimens were analyzed by a specialist in thoracic radiology and pathology. Third, we had long-term follow-ups with the patients. Our study's limitation is the small number of cases; however, we included a relatively large number of patients diagnosed by surgical lung biopsy compared with other studies that included patients diagnosed by PCNB or TBLB [9, 10, 13].

In conclusion, AFOP shows a high mortality rate of about 50%, but most of the survivors had normal life after treatment without dyspnea and supplemental oxygen therapy. Further large-scale studies are needed to better understand clinical course and find the prognostic factors of AFOP.

## Data Availability

The datasets used or analysed during the current study are included in this published article and supplementary file.

## Abbreviations

AFOP: Acute fibrinous and organizing pneumonia
BAL: Bronchoalveolar lavage
CRP: C-reactive protein
CT: Computed tomography
GGO: Ground-glass opacity
PCNB: Percutaneous needle biopsy
PCR: Polymerase chain reaction
TBLB: Transbronchial lung biopsy
VV-ECMO: Venovenous extracorporeal membrane oxygenation

## References

[1] Beasley MB, Franks TJ, Galvin JR, Gochuico B, Travis WD (2002). Acute fibrinous and organizing pneumonia: a histological pattern of lung injury and possible variant of diffuse alveolar damage. Arch Pathol Lab Med.

[2] Travis WD, Costabel U, Hansell DM, King TE, Lynch DA, Nicholson AG, Ryerson CJ, Ryu JH, Selman M, Wells AU (2013). An official American Thoracic Society/European Respiratory Society statement: update of the international multidisciplinary classification of the idiopathic interstitial pneumonias. Am J Respir Crit Care Med.

[3] Bhatti S, Hakeem A, Torrealba J, McMahon JP, Meyer KC (2009). Severe acute fibrinous and organizing pneumonia (AFOP) causing ventilatory failure: successful treatment with mycophenolate mofetil and corticosteroids. Respir Med.

[4] Arnaud D, Surani Z, Vakil A, Varon J, Surani S (2017). Acute fibrinous and organizing pneumonia: a case report and review of the literature. Am J Case Rep.

[5] Lu J, Yin Q, Zha Y, Deng S, Huang J, Guo Z, Li Q (2019). Acute fibrinous and organizing pneumonia: two case reports and literature review. BMC Pulm Med.

[6] Nguyen LP, Ahdoot S, Sriratanaviriyakul N, Zhang Y, Stollenwerk N, Schivo M, Harper R (2016). Acute fibrinous and organizing pneumonia associated with allogenic hematopoietic stem cell transplant successfully treated with corticosteroids: a two-patient case series. J Investig Med High Impact Case Rep.

[7] Ishiwata T, Ebata T, Iwasawa S, Matsushima J, Ota S, Nakatani Y, Tsushima K, Tada Y, Tatsumi K, Takiguchi Y (2017). Nivolumab-induced acute fibrinous and organizing pneumonia (AFOP). Intern Med.

[8] Wang Y, Zhao S, Du G, Ma S, Lin Q, Lin J, Zheng K, Zhang G, Matucci-Cerinic M (2018). Acute fibrinous and organizing pneumonia as initial presentation of primary Sjogren's syndrome: a case report and literature review. Clin Rheumatol.

[9] Dai JH, Li H, Shen W, Miao LY, Xiao YL, Huang M, Cao MS, Wang Y, Zhu B, Meng FQ (2015). Clinical and radiological profile of acute fibrinous and organizing pneumonia: a retrospective study. Chin Med J (Engl).

[10] Gomes R, Padrao E, Dabo H, Soares Pires F, Mota P, Melo N, Jesus JM, Cunha R, Guimaraes S, Souto Moura C *et al*: Acute fibrinous and organizing pneumonia: a report of 13 cases in a tertiary university hospital. *Medicine (Baltimore)* 2016, 95(27):e4073. 10.1097/MD.0000000000004073 PMC5058823 27399094

[11] Kang HJ, Choi SM, Jeong YJ, Park JS, Lee SW, Yoon HI, Lee JH, Lee C-T, Cho Y-J (2011). Severe acute fibrinous and organizing pneumonia with acute respiratory distress syndrome. Tuberc Respir Dis.

[12] Paek D, Koh Y, Park DU, Cheong HK, Do KH, Lim CM, Hong SJ, Kim YH, Leem JH, Chung KH *et al*: Nationwide study of humidifier disinfectant lung injury in South Korea, 1994–2011. Incidence and dose-response relationships. *Ann Am Thorac Soc* 2015, 12(12):1813–1821. 10.1513/AnnalsATS.201504-221OC 26653190

[13] Onishi Y, Kawamura T, Higashino T, Mimura R, Tsukamoto H, Sasaki S: Clinical features of acute fibrinous and organizing pneumonia: an early histologic pattern of various acute inflammatory lung diseases. *PLoS One* 2021, 16(4):e0249300. 10.1371/journal.pone.0249300 PMC8016294 33793625

